# Ultrasound stimulation improves inflammatory resolution, neuroprotection, and functional recovery after spinal cord injury

**DOI:** 10.1038/s41598-022-07114-6

**Published:** 2022-03-07

**Authors:** Yu-ri Hong, Eun-hee Lee, Ki-su Park, Mun Han, Kyoung-Tae Kim, Juyoung Park

**Affiliations:** 1 Medical Device Development Center, Daegu-Gyeongbuk Medical Innovation Foundation, Daegu, 41061 Korea; 2 Department of Neurosurgery, School of Medicine, Kyungpook National University Hospital, Kyungpook National University, 130 Dongdeok-ro, Jung-gu, Daegu, 41944 Korea; 3 Department of High-Tech Medical Device, Gachon University, Seongnam, 1342 Korea; 4 SonoTx, Seongnam, Korea

**Keywords:** Diseases, Health care, Neurology

## Abstract

Spinal cord injury (SCI) is associated with limited functional recovery. Despite advances in neuroscience, realistic therapeutic treatments for SCI remain unavailable. In this study, the effects of non-invasive ultrasound (US) treatment on behavior and inflammatory responses were evaluated in a rat model of SCI. Adult female Sprague–Dawley rats were subjected to spinal cord contusion injury. Two different US parameters (SCIU5: 5% and SCIU40: 40% duty cycle) were applied, and their effects on behavioral recovery after SCI were quantified. Tissue and neuronal responses were detected. Immunofluorescence was used to detect inflammatory markers. In the rat model of SCI, motor function was more effectively restored, and the lesion cavity area was smaller in the SCIU5 group. Furthermore, the SCIU5 protocol elicited an anti-inflammatory response at the injury site by reducing degenerative FJC-labeled neurons, macrophage/microglia activation, and infiltration. Thus, the lesion area decreased, and tissue density increased. Meanwhile, the SCIU40 protocol did not improve motor function or induce an anti-inflammatory response at the injury site. The SCIU5 protocol effectively accelerated the rate of improved exercise performance in the rat model while reducing inflammation. Accordingly, appropriate US stimulation may represent a promising treatment modality for SCI with beneficial anti-inflammatory effects.

## Introduction

Spinal cord injury (SCI) is associated with the severe and permanent impairment of motor and sensory functions. Functional deficits are induced by an initial mechanical injury and subsequent secondary injury lasting weeks or months^1–3^. Secondary injuries occur via a cascade of biochemical and pathological changes that exacerbate tissue damage, including inflammatory cell infiltration, oxidative stress, ischemia, inflammatory cytokine release, and initiation of apoptotic signaling cascades^4,5^. Therefore, therapeutic approaches should focus on early phase treatment to prevent damage induced by secondary pathophysiological processes. Several SCI treatment approaches have been evaluated, including surgical interventions^6^, pharmacological approaches^7–9^, stem cell treatment^10,11^, biomaterials^12,13^, and functional electrical stimulation^14,15^. However, studies examining the efficacy of these therapeutic strategies remain limited, and their application is highly debated owing to potential side effects and safety concerns^16–18^. Therefore, the development of effective, simple, and stable therapies for SCI is urgently needed.

The therapeutic application of ultrasound (US) has progressed substantially since the 1950s^19–21^. US energy deposited in tissues can induce biological effects via radiation and mechanical stress. US, alone or combined with microbubbles, is widely used for the treatment of various malignant human cancers^22–24^, drug delivery^25^, and curative therapies^26^. Moreover, several recent clinical trials and experimental studies have reported that US can elicit an anti-inflammatory response, promote tissue repair, and reduce pain^27–30^. US stimulation reduces inflammatory mediators, such as cyclooxygenase (COX)-1/2, inducible nitric oxide synthase (iNOS), tumor necrosis factor (TNF)-α, and interleukin (IL)-1β, as well as immune cell infiltration, in numerous inflammatory diseases, including peripheral nerve injury^31–33^. In particular, Zachs et al. demonstrated that daily US stimulation targeting the spleen could induce an anti-inflammatory response via immunomodulation of both T and B cells, conferring protective and therapeutic effects in inflammatory arthritis^34^. The non-invasive modulation of specific neuronal signaling pathways within the spleen circuit demonstrates the potential therapeutic benefits of US for inflammatory diseases. Furthermore, preclinical studies have reported transcranial US stimulation results in functional recovery and neuroprotective effects in neurodegenerative diseases via induction of brain-derived neurotrophic factor (BDNF)^35,36^. These results raise the possibility that US stimulation could block inflammatory reactions and neuronal damage while promoting neuronal regeneration.

In the current study, we investigated whether US stimulation has a therapeutic effect in an SCI rat model. We demonstrated that US stimulation improves locomotor behavior, reduces tissue/neuronal damage, and inflammatory responses by infiltrating immune cells. The observed anti-inflammatory effects support the potential clinical application of US to develop efficient and safe SCI treatments.

## Results

### Contusion force classification for optimal SCI induction

**Figure 1:**
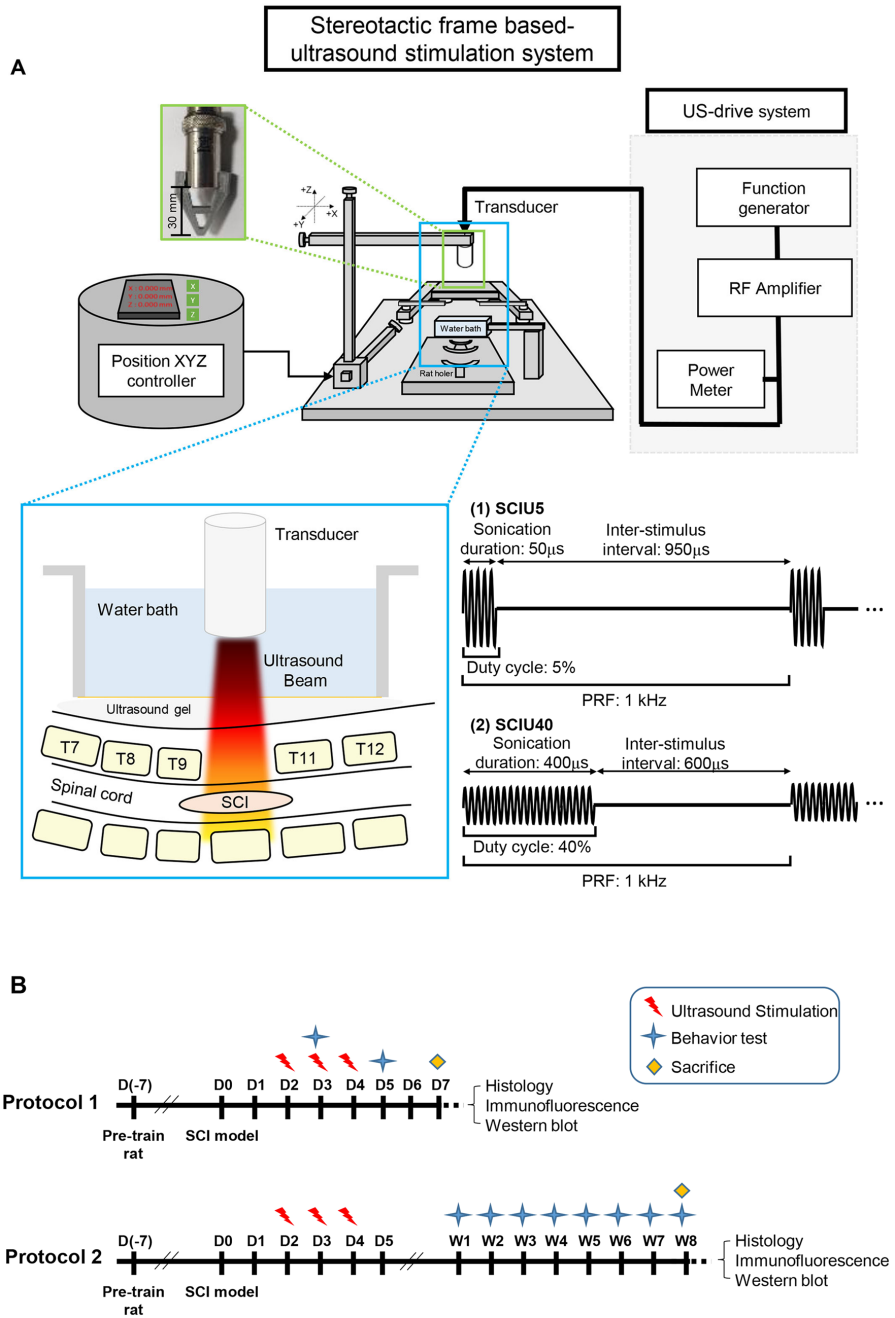
Experimental setup of the customized US stimulation system. (**A**) US stimulation with two different parameters, SCIU5 and SCIU40, was applied to spinal cord injury model rats. Schematic illustration of the customized US stimulation systems. (**B**) Experimental design for the evaluation of the effect of US stimulation on a rat model of SCI. Models were established on day 0 and subjected to US stimulation three times (2, 3, and 4 days following traumatic cord injury. Protocol 1; acute SCI phase study (days 1–7). Protocol 2; chronic SCI phase study (8 weeks).

Various degrees of force, ranging from 140 to 200 Kdyn, were evaluated (Fig. 1 and Table 1) to obtain a moderate injury level that allows reliability between Sham vs. US treatment in both acute and chronic post-SCI phases (Table 1 and Supplementary Fig. 1). In rats administered 150 and 200 Kdyn, the average Basso, Beattie, and Bresnahan (BBB) score was 0.3 ± 0.5 (*P* = 0.008) on day 5 after the injury, while that for 140 Kdyn was 3.4 ± 2.8. After 7 days, the mean BBB scores for 150 and 200 Kdyn were 0.8 ± 0.8 (*P* < 0.0001) and 1.3 ± 1.0 (*P* < 0.0001), respectively, which differed significantly from that for 140 Kdyn (i.e., 8.5 ± 2.2). Initial studies revealed that 140 Kdyn was an intermediate level of exposure at the T10 level of the spinal cord. Rats administered 150 and 200 Kdyn were nearly unable to perform locomotion even after weeks. We expected that the 140 Kdyn is more suitable for a moderate injury level that is enough to distinguish acute/chronic locomotor differences during the experiment. Therefore, 140 Kdyn injury level was used for subsequent analyses.

**Table 1:** Infinite Horizon impact parameters.

	Force (Kdyn)	Velocity (mm/s)	Time (ms)	Animal number
140 Kdyn	140 ± 7	122 ± 5	0	7
150 Kdyn	152 ± 2	126 ± 2	0	8
200 Kdyn	214 ± 20	119 ± 5	0	8

### Improvement of locomotor function during the acute SCI phase

**Figure 2:**
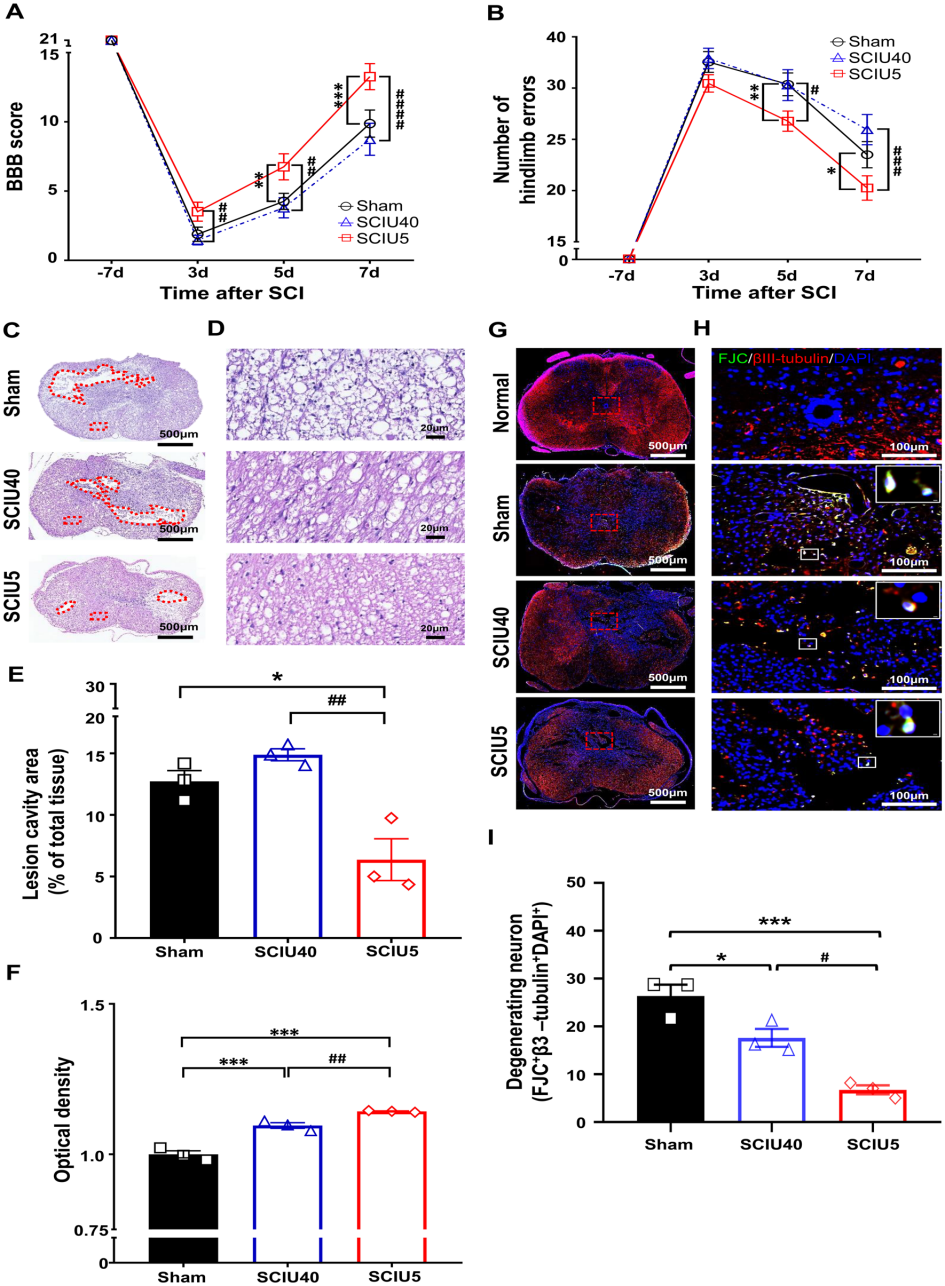
Improved function and reduced tissue damage in the acute spinal cord injury (SCI) phase. (**A**) The Basso, Beattie, and Bresnahan (BBB) score for hindlimb locomotor function was evaluated at 3, 5, and 7 days after US treatment post-SCI (black line indicates the sham group, n = 21; red line indicates the SCIU5 group, n = 21; blue line indicates the SCIU40 group, n = 18). (**B**) Fine motor coordination was evaluated using the ladder rung test by counting error steps. An error was defined as a slip, miss, or drag. (**C**, **D**) Representative images of spinal cord transverse sections for H&E staining from each group and enlarged images. (**E**) Quantification of the total cavity area, expressed as percentage of total spinal cord tissues following contusion injury (n = 3 per group). (**F**) The tissue density was assessed in H&E-stained slices from the regions in the ventral white matter, excluding the necrotic zone (n = 3 per group). (**G**) βIII-tubulin^+^/FJC^+^/DAPI^+^ staining and enlarged images at high magnification. Red square boxes indicate areas of gray matter adjacent to the injury region (Scale bar, 100 μm). White-boxed insets are higher-magnified views with βIII-tubulin^+^/FJC^+^/DAPI^+^ cells, which were counted as degenerating neurons (Scale bar, 2 μm). (**H**) Quantitative analysis of βIII-tubulin^+^/FJC^+^/DAPI^+^ cells expressed based on cell counting in gray matter. The bar graph shows the number of triple positive cells (shown as white signal) from the six ROIs adjacent to the lesion epicenter in the gray matter from three slices from three rats, based on non-overlapping fields (n = 3 per group, average values from the individual data points of ROIs). Data are expressed as means ± SEM. Two-way ANOVA with Turkey’s tests for the multiple comparisons was used. *P < 0.05, **P < 0.01, ***P < 0.001, and ****P < 0.0001 compared with the sham control, ^*##*^*P* < 0.01, ^*###*^*P* < 0.001, ^*####*^*P* < 0.0001 compared with the SCIU5-treated group.

We first investigated US treatment effects in the acute SCI phase (Table 2 and Supplementary Fig. 2). Following US stimulation for 2 to 4 days, functional motor recovery was assessed by BBB scores and the ladder rung test in both the SCIU5 and SCIU40 groups. Although scores were very low 3 days after SCI, slight differences were observed among the three experimental groups even at this early time point. The BBB score for the SCIU5 treatment group was 3.5 ± 3.2 compared to 1.9 ± 2.4 (*P* = 0.1) for the sham control and 1.5 ± 1.5 (*P* = 0.04) for the SCIU40 treatment group (Fig. 2a). At 5 and 7 days after spinal contusion, the BBB scores for the SCIU5-treatment group were 6.8 ± 2.5 and 13.3 ± 2.5, respectively, which differed significantly from those in the sham control group (4.3 ± 1.5 (*P* = 0.007) and 9.9 ± 2.6 (*P* = 0.0002), respectively). Meanwhile, on days 5 and 7 post-injury, the SCIU40 treatment group had BBB scores of 3.8 ± 1.8 (*P* = 0.8) and 8.7 ± 2.8 (*P* = 0.4), similar to those in the sham control group. Similarly, the average number of hindlimb errors in the SCIU5 treatment group improved significantly to 20.3 ± 5.3 mistakes compared to the sham control (23.5 ± 5.7 mistakes, *P* = 0.04) and SCIU40 treatment group (25.9 ± 6.3 mistakes, *P* = 0.0003), at 7 days (Fig. 2b). These results suggest that US treatment under the SCIU5 condition could improve hindlimb motor function recovery after SCI. In the SCIU40 group, hind leg motor function recovered similar to the sham control group.

**Table 2:** Summary of the experimental ultrasound (US) parameters.

Protocol	Group	US treatment condition	Center frequency (MHz)	Pulse repetition frequency (kHz)	Acoustic intensity (W/cm^2^)	Duty cycle (%)	Animal number
1	1	Sham	–	–	–	–	7
	2	SCIU5	1	1	0.8	5	8
	3	SCIU40	1	1	0.8	40	7
2	1	Sham	–	–		–	21
	2	SCIU5	1	1	0.8	5	21
	3	SCIU40	1	1	0.8	40	18

### Reduction of tissue and neuronal cell damage in the acute SCI phase

Lesion cavity volumes were assessed in serial hematoxylin and eosin (H&E)-stained spinal cord sections in the transverse plane after 7 days. The percentage of lesion cavity area was 6.4 ± 2.9% in the SCIU5-treated group and 12.7 ± 1.5% in the SCIU40-treated group (Fig. 2c,e). The lesion cavity area showed a significant decrease in the SCIU5-treated group. The tissue densities around the lesion were significantly higher in the SCIU5 and SCIU40 groups than in the sham control group (Fig. 2d,f). These results were confirmed by in vivo T2W MR images to identify structural changes at the spinal cord lesion site corresponding to histological features (Supplementary Fig. 3). At the lesion center, signal intensity at the lesion center, representing atrophy caused by hemorrhage, edema, and fibroglial scarring, was lower in the SCIU5-treated group than in the sham control, and SCIU40-treated group 14 days after SCI. We also observed a demyelinating lesion in the injured site following US stimulation. The myeline content significantly increased in the SCIU5-treated group compared to the sham control group and SCIU40-treated group in the white matter region (Supplementary Fig. 4).

To determine whether US stimulation could decrease neuron degeneration, we conducted double-staining immunofluorescence with microtubule-associated localization of βIII-tubulin and FJC. Co-staining of the tissue sections with βIII-tubulin (a neuronal cell marker) and FJC (neuronal damaged cell marker) showed the degenerative neuronal cells after contusion injury. The triple-positive signals of βIII-tubulin^+^/FJC^+^/DAPI^+^ showed a dramatic decrease in the injured tissues at the acute stage. The SCIU5 and SCIU40-treated group had a significantly lower number of degenerating neuronal cells compared to that in the sham control (Fig. 2g–i). These results suggest that SCIU40 treatment tends to increase nerve damage and that SCIU5 treatment has an inhibitory effect on tissue and nerve damage in acute SCI.

### Suppression of the inflammatory response in acute SCI

**Figure 3:**
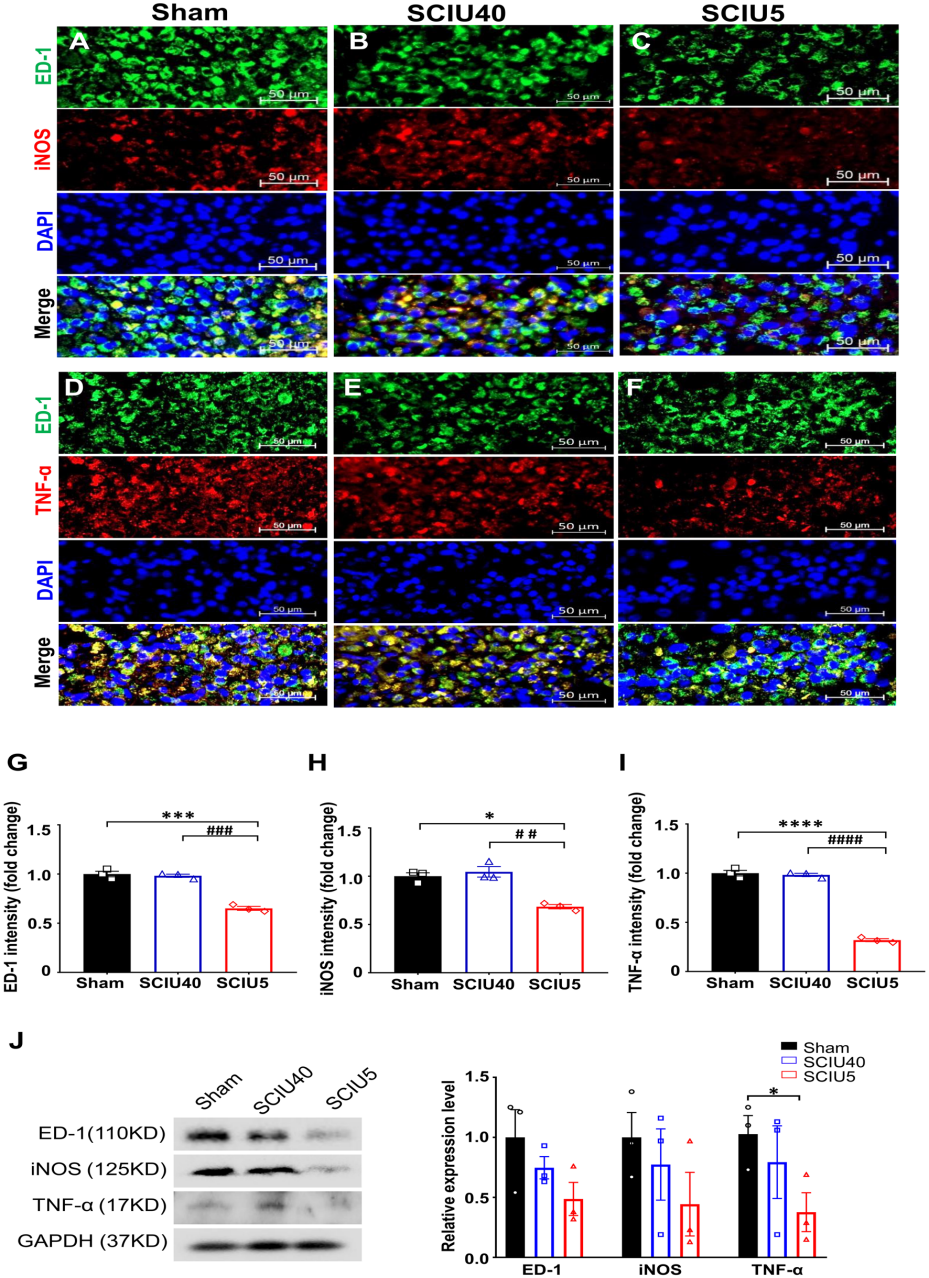
Ultrasound (US) stimulation reduces ED-1, iNOS, and TNF-α expression in the acute spinal cord injury (SCI) phase. (**A**–**C**) ED-1 (macrophage/microglia, green) and iNOS (inducible nitric oxide synthase, red) were evaluated in the sham (**A**), SCIU40 (**B**), and SCIU5 (**c**) experimental groups at 7 days post-SCI (five sections per rat, n = 3). (**D**–**F**) Cross-sections were counterstained with anti-TNF-α (red) and ED-1 (green) antibodies (five sections per rat, n = 3). (**G**–**I**) Quantification of fluorescence intensity of ED-1, iNOS, and TNF-α. Two-way ANOVA with Turkey's test for multiple comparisons was used for analyses. *P < 0.05, ****P < 0.0001 compared with the sham control, ^*#*^*P* < 0.05, ^*###*^*P* < 0.001, ^*####*^*P* < 0.0001 compared with the SCIU5-treated group. (**J**) Representative western blotting results for ED-1, iNOS, TNF-α, and GAPDH at 7 days post-SCI (Lane 1, sham; lane 2, SCIU40; lane 3, SCIU5;). Relative expression levels of ED-1, iNOS, and TNF-α were calculated after normalization to the level of GAPDH. Full-length blots are presented in Supplementary Fig. 8. Data are presented as means ± SEM. Two-tailed Student’s *t*-tests were used for comparison. *P < 0.05 compared with the sham control.

To investigate the effect of US treatment on the inflammatory response after SCI, immunofluorescent detection of ED-1 (macrophage/microglial cell marker), iNOS, and TNF-α was performed to determine the level of macrophage/microglial cell activation (Fig. 3, Supplementary Figs. 5, 7). The relative fluorescence intensity of ED-1-positive macrophages/microglia was low in the SCIU5-treated group compared with the sham control group 7 days after spinal contusion (0.65 ± 0.3-fold change, *P* = 0.01; Fig. 3g). Furthermore, levels of iNOS and TNF-α, indicators of the macrophage-induced inflammatory response, were substantially reduced in the SCIU5-treated group, with relative changes of 0.7 ± 0.2-fold (*P* = 0.1) and 0.3 ± 0.3-fold (*P* < 0.0001) compared to the sham control group (Fig. 3h,i). Meanwhile, the SCIU40 treatment group showed no differences in iNOS and TNF-α levels compared to those in the sham control group (fold changes: 1.1 ± 0.5 (*P* = 0.6) and 1.0 ± 0.3 (*P* < 0.9), respectively). These findings were confirmed by western blot analysis of pro-inflammatory cytokines in whole damaged tissues (Fig. 3j). In the SCIU5- treated group, the expression levels of ED-1, iNOS, and TNF-α were dramatically lower than those in the sham control and SCIU40-treated groups. Similarly, expression levels were lower in the SCIU40-treated group than in the sham control group. To assess whether chemokine expression regulated by US stimulation was caused by infiltration of peripheral macrophages, we performed a real-time qPCR to determine the expression level of the CCL2 and CCL5 on day 7 post-SCI. We showed that the level of CCL2 and CCL5 were significantly decreased in the SCIU5-treated group compared to that in the Sham control group (Supplementary Fig. 6). These results indicate that SCIU5 treatment has an inhibitory effect on the production of pro-inflammatory factors caused by the inflammatory response and immune cell infiltration in the acute injury stage.

### Locomotor recovery in the chronic SCI phase

**Figure 4:**
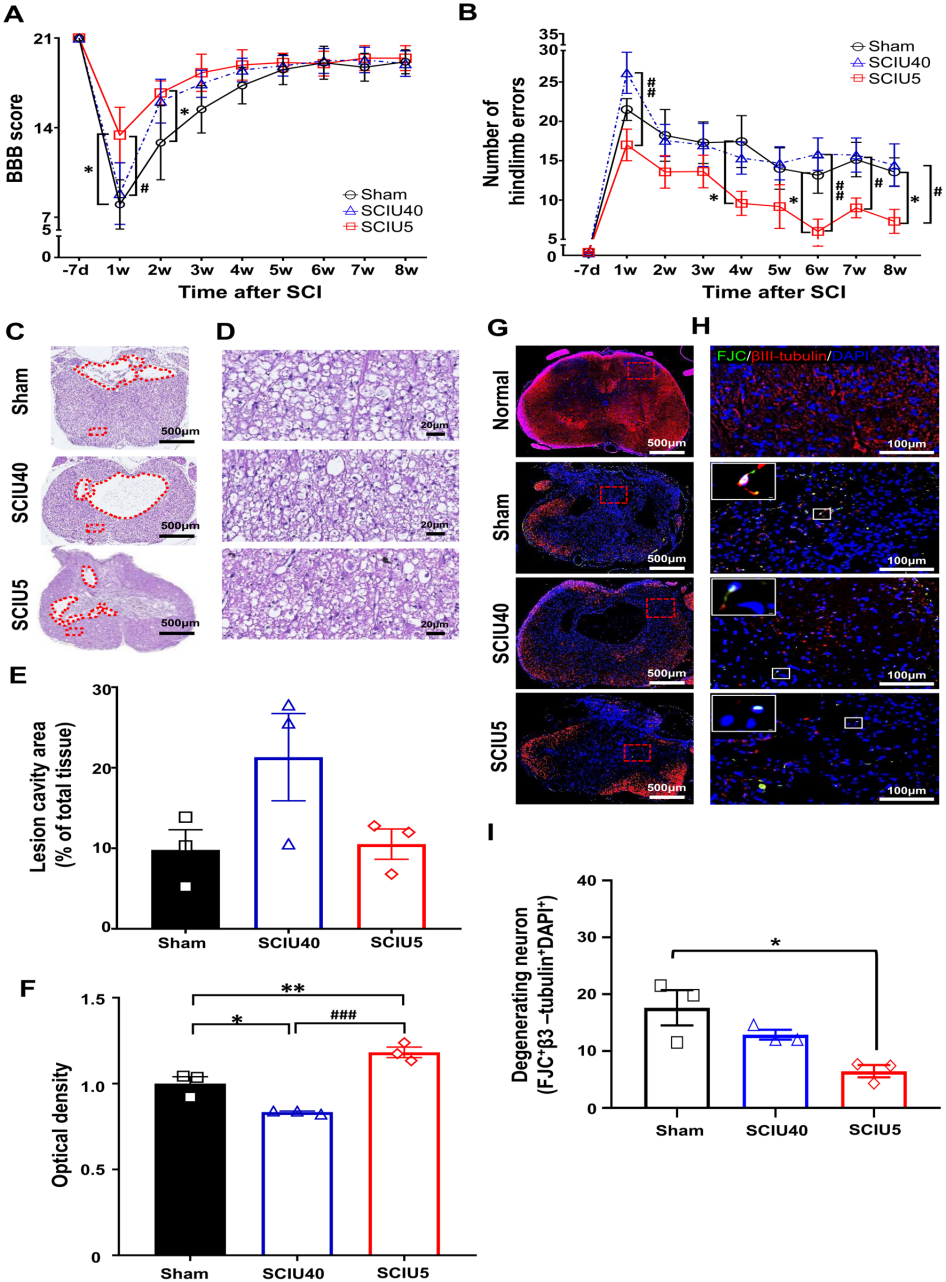
Ultrasound (US) stimulation significantly improves motor function and suppresses tissue damage in the chronic spinal cord injury (SCI) phase. (**A**) Basso, Beattie, and Bresnahan (BBB) scores for hindlimb locomotor function evaluated 7 days before (− 7) and 1–8 weeks after US stimulation post-SCI (black line indicates the sham group, n = 7; red line indicates the SCIU5 group, n = 8; blue line indicates the SCIU40 group, n = 7). (**B**) Fine motor coordination was evaluated using the ladder rung test based on the percentage of errors, defined as a slip, miss, or drag. (**C**, **D**) Transverse spinal cord sections obtained 8 weeks after SCI were stained with H&E to evaluate the area of tissue injury and cavity. The injured cavity area is marked with a dashed red line, and the red box in C is magnified 20 × in D. (**E**) The lesion cavity area of total tissue following contusion injury was quantified (n = 3 per group). (**F**) The tissue density was assessed in H&E-stained slices from the regions in the ventral white matter, excluding the necrotic zone (n = 3 per group) (**G**) βIII-tubulin^+^/FJC^+^/DAPI^+^ staining in transverse sections of the spinal cord and the enlarged images at high magnification (red square box, gray matter close to the lesions, Scale bar, 100 μm; white square box, βIII-tubulin^+^/FJC^+^/DAPI^+^ cells, Scale bar, 2 μm). (**H**) Quantitative analysis of βIII-tubulin^+^/FJC^+^/DAPI^+^ cells expressed based on cell counting in gray matter. The bar graph shows the number of triple positive cells (shown as white signal) from the six ROIs adjacent to the lesion epicenter in the gray matter from three slices from three rats, based on non-overlapping field (n = 3 per group, average values from the individual data points of ROIs). Data are expressed as means ± SEM. Two-way ANOVA and Turkey’s tests for the multiple comparisons were used. *P < 0.05, ***P < 0.001, ****P < 0.0001 compared with the sham control, ^*#*^*P* < 0.05, ^*##*^*P* < 0.01, ^#*###*^*P* < 0.0001 compared with the SCIU5-treated group.

Given the therapeutic effects of US treatment in the acute SCI phase, we tested whether this approach could also lead to locomotor function recovery in the chronic SCI phase. Functional locomotor tests were performed once per week for 8 weeks. (Fig. 1b, Protocol 2). Hind limb locomotor activity showed rapid recovery during the first 3 weeks and continued thereafter at a slower rate, with a BBB score similar to that of normal rats (i.e., 21). During the recovery period, the US-treated groups showed significantly improved motor function compared to the sham control group in the open-field test (Fig. 4a). One week later, the BBB scores in the SCIU5-treated group were 13.4 ± 3.1, whereas scores in the SCIU40-treated group (8.9 ± 3.1, *P* = 0.03) and sham control group (8.0 ± 3.2, *P* = 0.02) were > 5 points lower. Two weeks after spinal injury, the SCIU5- and SCIU40-treated groups had BBB scores of 16.7 ± 1.5 (*P* = 0.04) and 16.1 ± 2.6 (*P* = 0.1), respectively, which was higher than that of the sham control (12.8 ± 4.4). In the horizontal ladder rung walking test, the SCIU5-treated group showed significantly improved recovery of hindlimb motor activity, whereas the SCIU40-treated group showed an error similar to that of the sham control group until 8 weeks after SCI (Fig. 4b). These results suggest that US treatment can improve hindlimb motor function in the chronic SCI phase.

### Reduction of tissue and neuronal cell damage in the chronic SCI phase

To investigate the positive therapeutic effect on tissue damage 8 weeks after treatment, lesion cavity and neuronal cell death were assessed histologically. Upon histological evaluation, the percentage of lesion cavity area was 10.5 ± 1.9% in the SCIU5-treated group and 9.8 ± 2.5% in the sham control group. There was no statistically significant difference between the US treatment and sham control groups in the chronic SCI phase. Tissue density was 1.2 ± 0.3-fold (*P* < 0.01) higher in the SCIU5-treated group than in the sham control group. In contrast, in the SCIU40-treated group, the lesion cavity occupancy rate was higher, and the tissue density was lower by 0.8 ± 0.0-fold (*P* < 0.05) than those in the sham control group (Fig. 4c–f). We found that the myelination portion after injury was not significantly different in the experimental group in chronic stages of SCI (Supplementary Fig. 4). Furthermore, we analyzed βIII-tubulin^+^/FJC^+^/DAPI^+^-triple-positive neural cell damage. The triple-positive cells in the SCIU5-treated group were significantly lower than that in the sham control (Fig. 4g–i). We did not find a significant reduction in the lesion cavity area or neuronal cell death in the SCIU40 treatment group. Nevertheless, these results strongly suggest that SCIU5 therapy can be employed to prevent further damage to spinal nerve dysfunction, even in the chronic stage of SCI.

## Discussion

US stimulation can induce several biological effects mediated by radiations and mechanical stress. Recently, preclinical studies have reported that US application results in functional recovery and has neuroprotective effects in neurodegenerative and traumatic diseases via modulation of inflammation and neuroregeneration processes (via BDNF)^35,36^. This study showed that low-intensity US stimulation could induce the recovery of hindlimb dyskinesia in rats with a damaged spinal cord. Two US treatment conditions (SCIU5 and SCIU40) were evaluated to confirm their effectiveness. The SCIU5 treatment group showed a significant improvement in functional motor outcomes, supported by significant improvements in the BBB score and ladder rung test in the acute SCI model (Fig. 2a,b). In particular, in the chronic SCI model (Fig. 4a,b), the SCIU5 treatment group maintained a significantly higher rate of functional motor improvement in the ladder rung test compared to those in other conditions up to 8 weeks of follow-up. The performance improvement in the ladder rung test might be related to the recovery of the spinal circuit communication between the supraspinal and spinal circuits, which is essential for performance in the ladder rung test^37^.

Similar to the motor function results, histological improvements in the SCIU5 treatment group were confirmed in our study. The lesion cavity size and neuronal cell damage in the SCIU5 treatment group were significantly decreased in the acute SCI model (Fig. 2c–i) and the chronic SCI model (Fig. 4c–i). Indeed, low-intensity pulsed US therapy has been reported to modulate cytokine production, phagocytosis, and the expression of genes related to tissue repair^38,39^. US treatment can also increase neurotrophic factor protein levels to suppress apoptosis and protect nerves in an animal model of traumatic brain injury^40^. Hence, the observed histopathological improvements could provide evidence of anti-inflammatory effects by inducing neurotrophic factors and tissue repair by US stimulation.

In previous studies, edema and neutrophil infiltration in a rodent model of foot edema were significantly reduced by low-intensity ultrasound stimulation^31,41^. Similarly, we observed the development of edema (Supplementary Fig. 3) and increased levels of inflammatory factors (ED-1, iNOS, and TNF-α; Fig. 3) within the SCI area, all of which was reduced following SCIU5 treatment. Furthermore, the group treated with SCIU5 showed decreased macrophage marker ED-1, indicating that the response to US stimulation was closely related to the regulation of inflammatory cell infiltration. The expression of iNOS is strongly linked to activated macrophages and accelerates tissue damage following SCI^42,43^. In the SCIU5 treatment group, iNOS expression was significantly reduced in the damaged spinal cord, indicating a prominent decrease in neurodegeneration and neuroinflammation. These raise the question regarding the mechanisms by which US stimulation is associated with recruiting other immune cells or altering the phenotype or function of macrophages. US stimulation could recruit monocyte-derived macrophages, astrogliosis, and additional immune subsets (i.e. T & B lymphocytes) to the microenvironment created by the lesion, and affect blood-spinal cord barrier integrity. Furthermore, it could affect the phagocytosis of macrophage or phenotypic switch of the macrophage/microglial polarization (M1/M2). Although the underlying mechanism remains to be determined, this study strongly suggests that US stimulation could play a pivotal role as an anti-inflammatory factor altering macrophages to be less damaging and more beneficial for functional recovery.

In the present study, we applied two US conditions US5 and US40, with different duty cycles. We used the abbreviation SCIU5 and SCIU40 to represent the corresponding percent of duty cycles. The peak pressure of SCIU5 (5% duty cycle) was 0.65 MPa at 1 MHz, and it fired 50 times in the treatment duration (per 1 ms). The SCIU40 (40% duty cycle) measured 0.22 MPa and fired 400 times in the same period. Although the US conditions had different duty cycles and produced different peak pressure amplitudes, both had an acoustic intensity of 0.8 W/cm^2^ (Supplementary Fig. 2). Recent studies have proposed that non-invasive US stimulation could elicit different neural modulative effects when stimulated with same intensity US having different parameters^44,45^. A low duty cycle (5%) US stimulation suppresses cortical neuron activity while a wave excites it. Thus, we speculated that SCIU5 (5% duty cycle mode) and SCIU40 (40% duty cycle mode), at the same intensity (0.8 W/cm2), would elicit neuronal modulation and inflammatory responses in SCI depending on the differential US parameters. We expected that the SCIU5 (a suppression US condition) could be more efficient on the inflammatory condition than the SCIU40 (an activation US condition). We also anticipated the possibility that SCIU40 could increase the neural motor activity in the lesion site of the SCI model. A low duty cycle (5%) US stimulation suppresses cortical neuron activity while a wave excites it. Thus, we speculated that SCIU5 (5% duty cycle mode) and SCIU40 (40% duty cycle mode), at the same intensity (0.8 W/cm^2^), would elicit neuronal modulation and inflammatory responses in SCI depending on the differential US parameters. We expected that the SCIU5 (a suppression US condition) could be more efficient on the inflammatory condition than the SCIU40 (an activation US condition). We also anticipated the possibility that SCIU40 could increase the neural motor activity in the lesion site of the SCI model. Here, our observations provide the first evidence that SCIU5suppresses the inflammatory response and enhances the locomotor functions, whereas SCIU40 did not significantly improve functional outcomes (Figs. 2, 4) or inflammatory responses (Fig. 3). Considering that we applied US treatment during the acute phase (2–4 days after SCI), which is associated with a massive inflammatory response^1^, it is reasonable to speculate that the suppressive US parameter induced by low duty cycles might be more effective than activation conditions when treating acute phase SCI.

Here, we performed the functional recovery assessment to address the effect of US stimulation following contusion injury. Although some spontaneous recovery was observed over the initial two-week period after injury in the chronic model, the BBB score for the SCIU5 treatment group showed a significant improvement after 1–2 weeks in the chronic phases. However, the final BBB scores are the same at 8 weeks in the chronic stages for all groups. As with BBB locomotor rating, we also demonstrated a significant improvement in the performance of the hindlimb using the horizontal ladder lung test. In addition, we further performed histological evaluation, ED-1 positive macrophage, and level of iNOS & TNF-α, suggesting that US stimulation could suppress the inflammation at the acute phase (Figs. 2, 3, 4). Moreover, we determined that the expression level of CCL2 and CCL5 chemokine was decreased in the SCIU5-treated group in the acute stage (Supplementary Fig. 6). It is believed that the beneficial effects of the US treatment stimulation could be attributed to the inhibition of acute inflammatory response in the injury site. Although the possibility could be raised that US treatment enhances activation of spinal motor pathways caudal to the injury site, we considered that the effective parameter of SCIU5 (5% duty cycle mode, neuronal suppressive effects) is insufficient to lead the motor neuronal activation. This view is linked to the expectation that the treatment with other US paradigms or combination stimulation with SCIU40 parameters could improve motor function recovery much better than a single US paradigm. Thus, the efficacy of additional US paradigms in providing long-lasting improvements will be evaluated in future studies.

Taken together, US treatment with the SCIU5 protocol could improve motor function while reducing inflammation and tissue damage in a rat model of SCI. These results suggest that US stimulation is a promising non-invasive treatment modality for SCI.

## Methods

### Animals

A total of 105 adult female Sprague–Dawley rats (weighing 200 ± 40 g; Orient Bio Inc., Seongnam, Korea) were used for all the experiments (Table 1). The study was approved by the Daegu-Gyeongbuk Medical Innovation Foundation (DGMIF) and the Institutional Animal Care and Use Committee (IACUC, DGMIF-19040101–00). Rats were maintained under specific pathogen-free conditions in individual ventilating systems; The rats were fed a normal pellet diet and water ad libitum. They were housed at a temperature of 22 ± 1 °C with a relative humidity of 50% ± 10%, using 12 h light/dark cycles, illumination at 150–300 Lux and ventilation 10–20 times/hour. Animals were anesthetized for all the experimental procedures and were constantly monitored throughout the course of the experiment. Animal handling and all the procedures were performed in accordance with the ethical guidelines for animal studies; the experiments were carried out in compliance with the appropriate Animal Research: Reporting In Vivo Experiments (ARRIVE) guidelines.

### Establishment of the SCI model

Rats were anesthetized with a combination of Zoletil 25 mg/kg (Virbac Laboratories, Carros, France) and Rumpun (4.6 mg/kg; Bayer, Leverkusen, Germany). The fur was then shaved from the backs of rats using a depilatory cream. Dorsal laminectomy was performed at the T10 level. Following laminectomy, each animal received kanamycin sulfate (0.05 mg/kg; Yuhan, Seoul, Korea). SCI was induced using an Infinite Horizon (IH) SCI device (Precision Systems & Instrumentation, Lexington, KY, USA)^46^. To evaluate the optimal contusion level for the long and short-term experiments, various degrees of force were evaluated from 140 to 200 Kdyn. The detailed parameters for the IH impactor are summarized in Table 1. After surgery, the animals were placed on a heating pad in a humidity- and temperature-controlled chamber. Bladders were manually evacuated twice daily until autonomous urination was established. Animals were randomly assigned to three experimental groups following surgery. All animals received the same SCI to reduce any bias. The rats were randomized into groups and identified using numbers written with permanent markers on their tails. During the follow-up behavioral testing, a blinded experiment was carried out with the experimental conditions of the individual animals blinded. The rats were euthanized by transcardiac perfusion at 7- and 56-days post-SCI.

### US stimulation system

The stereotactic frame-based US stimulation system (Fig. 1a) was composed of 3D-printed rat holders, a coupling water tank, a flat piezoelectric single-element transducer (A303S, f = 1 MHz, 12.7-mm diameter, Olympus America Inc., Waltham, MA, USA), a position XYZ controller (51900; Stoelting Inc., Wood Dale, IL, USA), a function/arbitrary waveform generator (33220A; Agilent Technologies, Santa Clara, CA, USA), and an RF power amplifier (A-150; Electronics & Innovation, Rochester, NY, USA). The center frequency of the transducer was 1 MHz. The US beam profile on the free field was measured using an acoustic intensity measurement system (AIMS III; ONDA, Sunnyvale, CA, USA) with a hydrophone (HGL-400; ONDA; Supplementary Fig. 1). The transducer adaptor apparatus was designed considering the beam profile, with a height of 30 mm, the focus point of ultrasonic energy.

### Experimental setup and US stimulation

Following anesthetization, SCI rats were placed on a 3D-printed rat holder (Fig. 1a). The US transducer was moved to the target position using an XYZ controller. The Z-axis, a constant distance between the transducer and the stimulation point, was adjusted using an inverted triangular 3D-printed transducer adaptor that clipped onto the transducer and allowed targeting. After obtaining the coordinates, the acquired values were set to zero. The water tank was placed at the target point of the dorsal laminectomy site after application of the ultrasonic gel. The transducer was positioned with its active element immersed in water, approximately 30 mm from the target region. All animals were subjected to two different stimulation conditions. The following pulse US parameters were used: (1) SCIU5, acoustic frequency of 1 MHz, pulse repetition frequency (PRF) of 1 kHz, 5% duty cycle, acoustic power of 0.8 W/cm^2^ and (2) SCIU40, acoustic frequency of 1 MHz, PRF of 1 kHz, 40% duty cycle, and acoustic power of 0.8 W/cm^2^. The total sonication time was 5 min (Table 2). Sham control rats were subjected to the same procedure as that of the experimental group; briefly, the mice were anesthetized and placed on the stereotactic frame with US gel for 5 min, but were not stimulated with US. These US parameters were based on the neuromodulation parameter ranges for excitatory or suppressive responses^47^.

### Experimental design

US stimulation was applied for 5 min daily from day 2 to day 4 (3 times total). The experimental protocol was divided into short-term (7 total) or long-term (8 weeks; Fig. 1b). The effects of US on locomotion and inflammation were assessed after SCI in three randomly assigned treatment groups: (1) sham (SCI only), (2) SCIU5 (SCI + US sonication, duty cycle 5%), and (3) SCIU40 (SCI + US sonication, duty cycle 40%). The detailed experimental groups and US parameters are described in Table 2.

### MR imaging

Images of rats were obtained 14 days post-injury. All the T-spine MRI scans were conducted on a 3 T clinical dedicated head MR scanner (Siemens Magnetom Skyra; Siemens Medical Solutions, Erlangen, Germany). A 40 mm loop-type RF coil was used, and rats were placed in the prone position under the coil housing. Sagittal images of the spine were acquired using 2D turbo spin-echo T2-weighted images. The acquisition parameters were as follows: field of view = 30 × 30 mm; matrix size = 128 × 128; number of slices = 10; slice thickness = 0.7 mm; no gap; repetition time = 2500 ms; echo time = 101 ms; number of averages = 20; echo train length = 10. MR images were assessed using Image J 1.52 (National Institutes of Health, Bethesda, MD, USA).

### Evaluation of locomotor function

Locomotor recovery after SCI was evaluated using the open-field BBB scale and ladder rung test. Two observers, blinded to the US treatment groups, evaluated animals based on the BBB open-field locomotion rating scale. The 21-point open-field locomotion score is based on the hindlimb locomotor ability of the SCI model and reflects the early (BBB score 0–7), intermediate (8–13), and late phases (14–21) of recovery^48^. After SCI rats briefly adapted to the conditions, they were observed in an open field for 5 min.

A horizontal ladder rung test was also used to evaluate hindlimb movements^49^. The ladder rung test apparatus consisted of sidewalls (length: 1 m, height: 50 cm) and metal rungs (diameter: 3 mm, length: 150 mm). Mistakes during walking on irregular runways were evaluated. During pretraining, the animals passed across the horizontal ladder twice. After familiarization, the SCI rats were recorded using a video camera. The hindlimb foot error was defined as a complete miss or slight/deep slip, and errors were counted. If an SCI rat could not take a step, it was assigned one error per bar, resulting in a total of 35 errors. All animals showed almost complete paraplegia 1 d after SCI. After the surgery, motor function was assessed on days 3, 5, and 7, then weekly for 8 weeks.

### Tissue preparation and histology

The rats were transcardially perfused with 100 mL of fresh 4% paraformaldehyde (PFA) in 1% phosphate-buffered saline (PBS; pH 7.4), followed by 100 mL of 1% PBS 7 days or 8 weeks after SCI. The injured spinal cords (approximately 6 mm) were dissected and post-fixed in PFA for 24 h at 4 °C. The tissues were embedded in paraffin blocks, and coronal sections were obtained. For H&E staining, the spinal cords were embedded in paraffin and serially sectioned at a 5-µm-thick coronal plane. Three to five representative section on every 60th slide corresponding to every 300 µm along the cord was used for histological experiments. All tissue slices were blinded to reduce bias. The tissue sections were deparaffinized with xylene, rehydrated in a series of ethanol (100%, 95%, 70%, and 50%), washed with distilled water (DW). To minimize bias, staining was performed after covering the name tag on the slides. All histological procedures were performed after mixing sections from different groups. Although it was not possible to achieve a completely blinded test, we attempted to remove any bias during the experiment.

### Immunofluorescence analysis

Next, 5-µm-thick transverse sections were blocked with PBS containing 1% BSA, 3% normal goat serum, and 0.4% Triton X-100 for 1 h at room temperature following the antigen retrieval process. Tissues were incubated at 4 °C in a humidified chamber overnight with primary antibodies anti-ED-1 (1:200; MAB1435; Merck, Kenilworth, NJ, USA), iNOS (1:200; #ab15323, Abcam, Cambridge, UK), and TNF-α1 (200; #ab6671; Abcam). Alexa Fluor 488-labeled goat anti-mouse-IgG (1:1000; #A32723, Invitrogen, Carlsbad, CA, USA), and Alexa Fluor 633-labeled goat anti-rabbit-IgG (1:1000; #A32731, Invitrogen) were used as secondary antibodies. After immunolabeling, the tissue slides were mounted in DAPI-containing fluorescence mounting medium (Dako).

### Image analysis

All tissue slides were captured using a Zeiss Axio Scan.Z1 Digital Slide Scanner (Carl Zeiss, Oberkochen, Germany) using a 20 × objective lens. Five slices stained with H&E from each animal were used to assess the lesion cavity volume. To evaluate tissue density, 4 regions of interest (ROIs) in the ventral white matter region per slide were selected and analyzed at high magnification (× 200). When selecting ROIs, areas except for the necrotic zone were obtained in the H&E-stained images to determine the tissue density resulting from hemorrhage and edema due to extensive spinal cord damage. To quantify immunofluorescence intensity, five serial sections in the transverse plane from each animal were analyzed using Image J (version 1.40; National Institutes of Health, Bethesda, MD, USA). Regions adjacent to the lesion in the immunofluorescence image were selected as the ROI.

### Double labeling with Fluoro-Jade C (FJC) and immunofluorescence

Double labeling with FJC and βIII-tubulin (a neuronal cell marker) was performed to detect degenerating neurons. Immunofluorescence staining was performed as described above. The tissue slides were incubated with mouse anti-βIII-tubulin (1:200; R&D system, # MAB1195) at 4 °C overnight, followed by incubation with Alexa Fluor 555 goat anti-mouse IgG (1: 1000, Cell Signaling, #.4409) for 1 h at room temperature. FJC staining was performed with the Biosensis’ ready-to dilute FJC staining kit (Biosensis, Tebarton, South Australia) according to the manufacturer’s protocol. Briefly, all fluorescently labeled slides were immersed in 10% potassium permanganate (v/v) for 5 min and then rinsed in distilled water for 2 min twice. Subsequently, the slides were incubated with 20% FJC and 20% DAPI (v/v) in 0.1% acetic acid solution for 20 min at room temperature. After washing with distilled water, the slides were dried and covered with a coverslip using DPX mounting media (Sigma-Aldrich).

### Western blotting

The SCI site was extracted and homogenized in RIPA lysis buffer containing protease inhibitor (150 mM sodium chloride, 0.1% SDS, 1% Triton X-100, 1% sodium deoxycholate, and 50 mM Tris–HCl). After centrifugation, the supernatant was transferred to a new tube. Protein concentration was determined using the Pierce BCA Protein Assay Kit (Thermo Fisher Scientific, Waltham, MA, USA). The proteins (40 µg/lane) were separated by 8–20% SDS-PAGE and transferred electrophoretically to a polyvinylidene difluoride membrane (Millipore, Billerica, MA, USA). After blocking with 5% skim milk, the membranes were incubated with an anti-iNOS mouse monoclonal antibody (1:200; #MAB9502, R&D Systems, Minneapolis, MN, USA), anti-TNF-α rat polyclonal antibody (1:500; #ARC3012, Invitrogen), anti-ED-1 mouse monoclonal antibody (1:200; #MAB1435, Merck), and anti-GAPDH antibody (1:10,000; #88845, Cell Signaling, Danvers, MA, USA), and subsequently incubated with the HRP-conjugated anti-rabbit-IgG secondary antibody (1:3000; #7074, Cell Signaling), and anti-rat-IgG secondary antibody (1:3000; #7077, Cell Signaling) for 2 h at room temperature. Signaling was detected using a chemiluminescence kit (Amersham Pharmacia Biotech Inc., Piscataway, NJ, USA). GAPDH was used as the loading control (Supplementary Fig. 8).

### Statistical analysis

All data are presented as means ± SEM. Statistical analyses and data visualization were performed using Prism version 6 (GraphPad Software, Inc., La Jolla, CA, USA). One- or two-way ANOVA and post-hoc Tukey’s multiple comparison tests were used for statistical analyses. Statistical significance was set at *P* < 0.05.

## Supplementary Information

Supplementary Information.

## Data Availability

Data are available from the corresponding author upon reasonable request.
