# Supplementary material for: Ultrasound stimulation improves inflammatory resolution, neuroprotection, and functional recovery after spinal cord injury
Source: Sci Rep. 2022 Mar 7;12:3636. doi: 10.1038/s41598-022-07114-6 (PMC8901758; doi:10.1038/s41598-022-07114-6)
Supplement: Supplementary file 1 — Supplementary Information. [file 41598_2022_7114_MOESM1_ESM.docx]

**Ultrasound stimulation improves inflammatory resolution, neuroprotection, and functional recovery after spinal cord injury**

Yu-ri Hong^1, †^, Eun-hee Lee^1, †^, Ki-su Park^2^, Mun Han^1^, Kyoung-Tae Kim^2,^ *, Juyoung Park^1, 3, 4^ *

^1^ Medical Device Development Center, Daegu-Gyeongbuk Medical Innovation Foundation, Daegu, Republic of Korea

^2^ Department of Neurosurgery, School of Medicine, Kyungpook National University, Kyungpook National University Hospital, Daegu, Korea

^3^ Department of High-tech medical device, Gachon University, Seongnam, 1342 Korea

^4^ SonoTx, Seongnam, Korea

^†^The authors contributed equally to this work.

*Corresponding authors:

Kyoung-Tae Kim, M.D., Ph.D., Department of Neurosurgery, School of Medicine, Kyungpook National University, Kyungpook National University Hospital, 130 Dongdeok-ro, Jung-gu, Daegu 41944, Korea, E-mail: [nskimkt7@gmail.com](mailto:nskimkt7@gmail.com)

Juyoung Park. Daegu-Gyeongbuk Medical Innovation Foundation, Medical Device Development Center, Daegu, 41061, South Korea; E-mail: [opedoors@gmail.com](mailto:opedoors@gmail.com)

**This file includes:**

Supplementary Methods

Supplementary Figure 1 to 8

**Supplementary Methods**

**Luxol Fast Blue (LFB) staining**

Coronal sections (5 μm thickness) were deparaffinized and rehydrated in a step-wise gradient. The sections were incubated with LFB staining solution at 60 °C, overnight. The dye was rinsed out with 95% ethanol and tap water sequentially, followed by differentiating with 0.05% lithium carbonate solution. This process was repeated 3 times in order to obtain clear differentiation between white matter and gray matter on monitoring under a microscope. After the differentiation procedure, the slides were placed into 0.5% Cresyl violet solution for 5 min at room temperature. After washing with tap water several times, the slides were dehydrated in ethanol twice and xylene twice. The slides were mounted with DPX mounting media and visualized under an Axio Scan.Z1 microscope. Myelin density was quantified by measuring the mean intensity of the red channel (IR) in the white matter and not in the gray matter[1](#_ENREF_1). The red channel is complementary to the blue channel saturation. LFB optical density (%) was calculated for each structure by 100% × [1 - (IR/256)], which yields a high myelin content for increasing values. The value was re-converted into relative myelin contents compared to normal tissue (% of normal tissue level). Individual mean intensity was obtained from three adjacent sections per location.

**Real-time quantitative PCR**

Total RNA from the spinal cord was extracted using the RNeasy Plus kit (Qiagen, Hilden, Germany) and cDNA synthesized using the Omniscript RT kit (Qiagen), according to the manufacturer’s instructions. The levels of gene expression were quantified by real-time quantitative PCR using SYBR® Premix Ex Tag™ II (Tli RNaseH Plus, Takara Bio Inc., Otsu, Shiga, Japan) and CFX96 Thermal cycler (Bio-rad, Hercules, CA, USA). The sequences of the primers were as follows: ccl2 (forward, 5′-GATGCAGTTAATGCCCCACT-3′; reverse, 5′-TTCCTTATTGGGGTCAGCAC-3′), ccl5 (forward, 5′- ATATGGCTCGGACACCACTC-3′; reverse, 5′CCCACTTCTTCTCTGGGTTG-3′), gapdh (forward, 5′- GGCATTGCTCTCAATGACAA-3′; reverse, 5′- TGTGAGGGAGATGCTCAGTG-3′). The gapdh gene was amplified separately as an internal control for normalization. The results were analyzed using Bio-Rad CFX96 software.


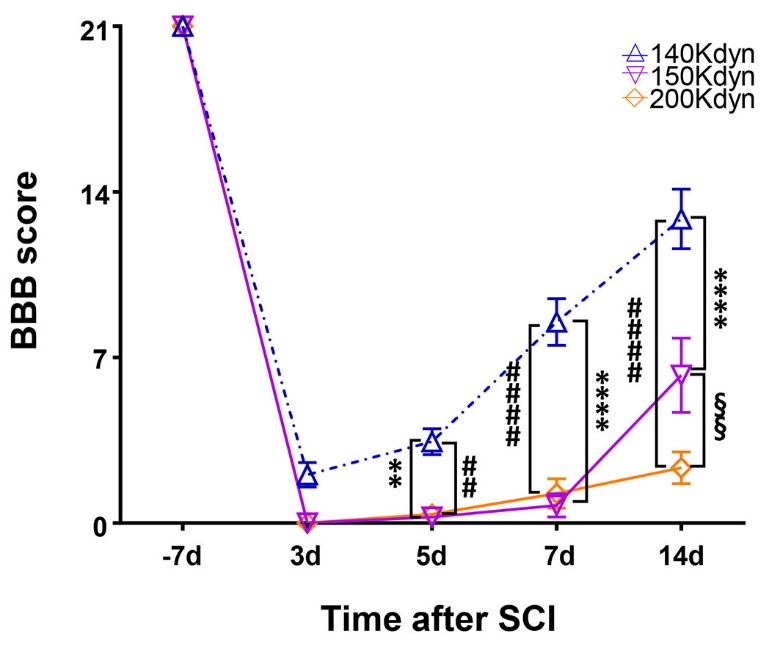


**Supplementary Figure 1. BBB score after SCI with various impact forces.** The 140 Kdyn group showed the greatest improvement in BBB scores over time. Seven days after SCI, the 140 Kdyn group (n = 7) showed a BBB score of 8–14 (intermediate recovery stage). When 150 (n = 8) and 200 Kdyn (n = 8) were applied, the BBB scores were 0–7. The difference between the 140 Kdyn group and 150 and 200 Kdyn groups was significant. Data are presented as means ± SEM. Two-way ANOVA and Tukey’s tests for multiple comparisons were performed. **P < 0.01, ****P < 0.0001 compared with 140 Kdyn, ##P < 0.01, ####P < 0.0001 compared with 150 Kdyn, and §§P < 0.01, compared with 200 Kdyn.


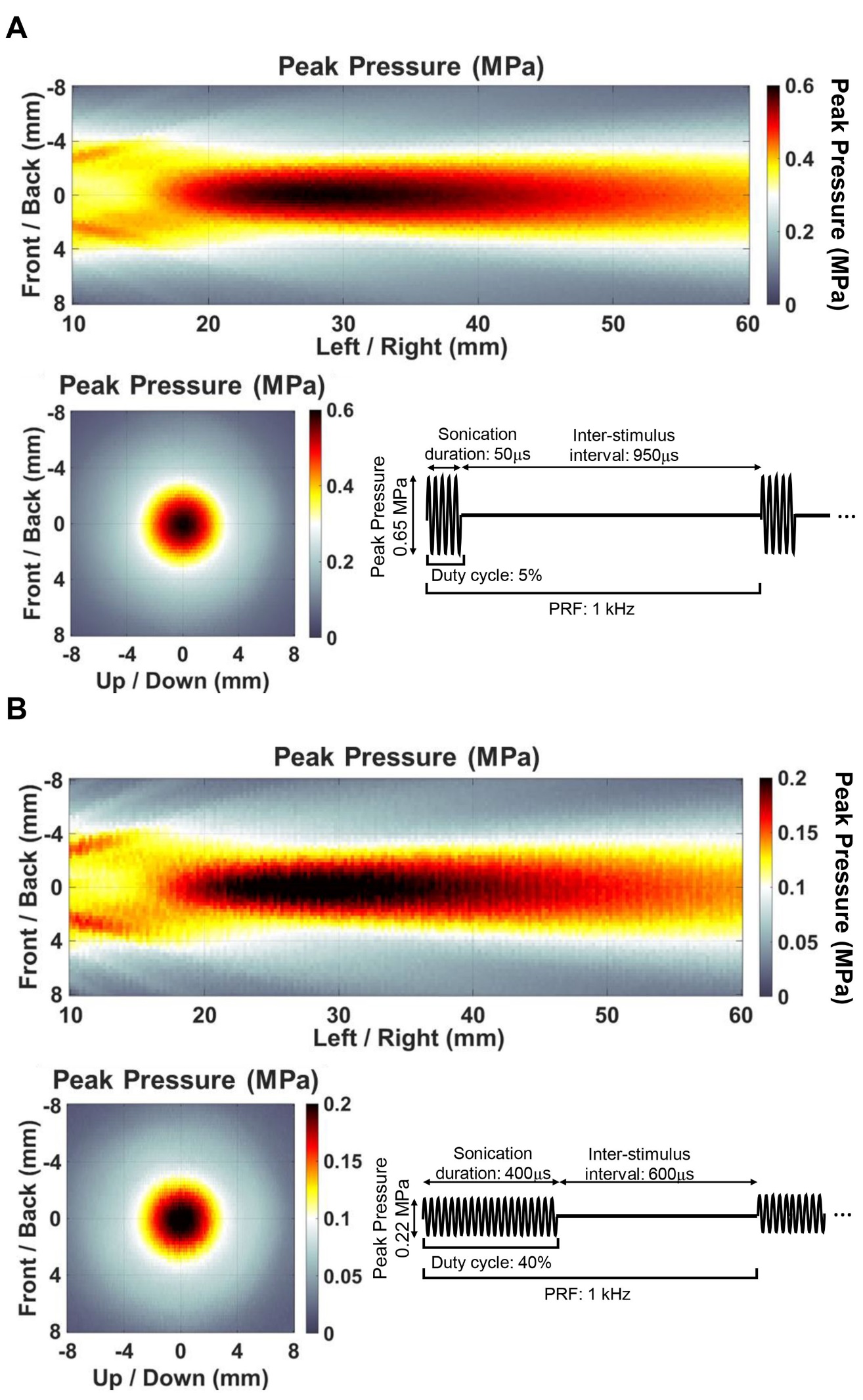


**Supplementary Figure 2. Peak acoustic pressure distribution.** Measurement of acoustic output distribution in a focal zone using the 1 MHz transducer in the lateral and axial planes. (a) SCIU5; duty cycle 5% and acoustic intensity of 0.8 W/cm^2^ (b) SCIU40; duty cycle 40% and acoustic intensity of 0.8 W/cm^2^. The maximum pressure at focus target was 0.65MPa (a) and 0.22MPa (b). The heat map scale represents the peak pressure (MPa). The data visualization was performed with MATLAB.


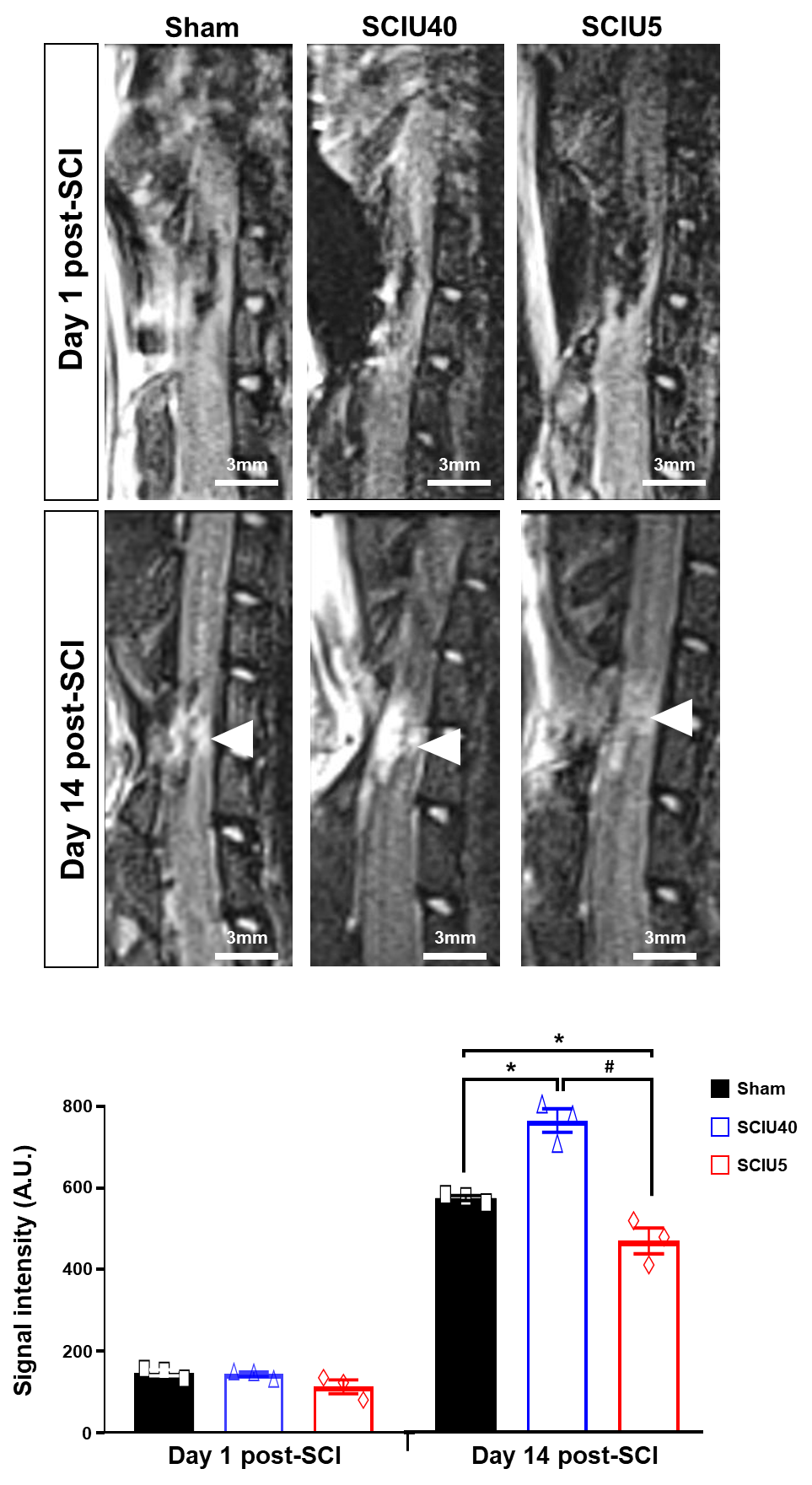


**Supplementary Figure 3. MR images (3.0 T) of the spinal cord lesions.** Sagittal T2-weighted MR imaging scans of the contused rat spinal cord were obtained in the sham (left), SCIU5 (middle), and SCIU40 (right) groups at 1 and 14 days after SCI. At 14 days, the SCI lesion epicenter (arrows) displayed lower signal intensity in the SCIU5 group than in the sham and SCIU40 groups. Scale bar = 3 mm. The T2 intensity was quantified using Image J software as the lesion intensity. Two-way ANOVA with Turkey's test for multiple comparisons was used for analyses. **P* < 0.05, compared with the sham control, *^#^P* < 0.05, compared with the SCIU5-treated group (n = 3/per group).


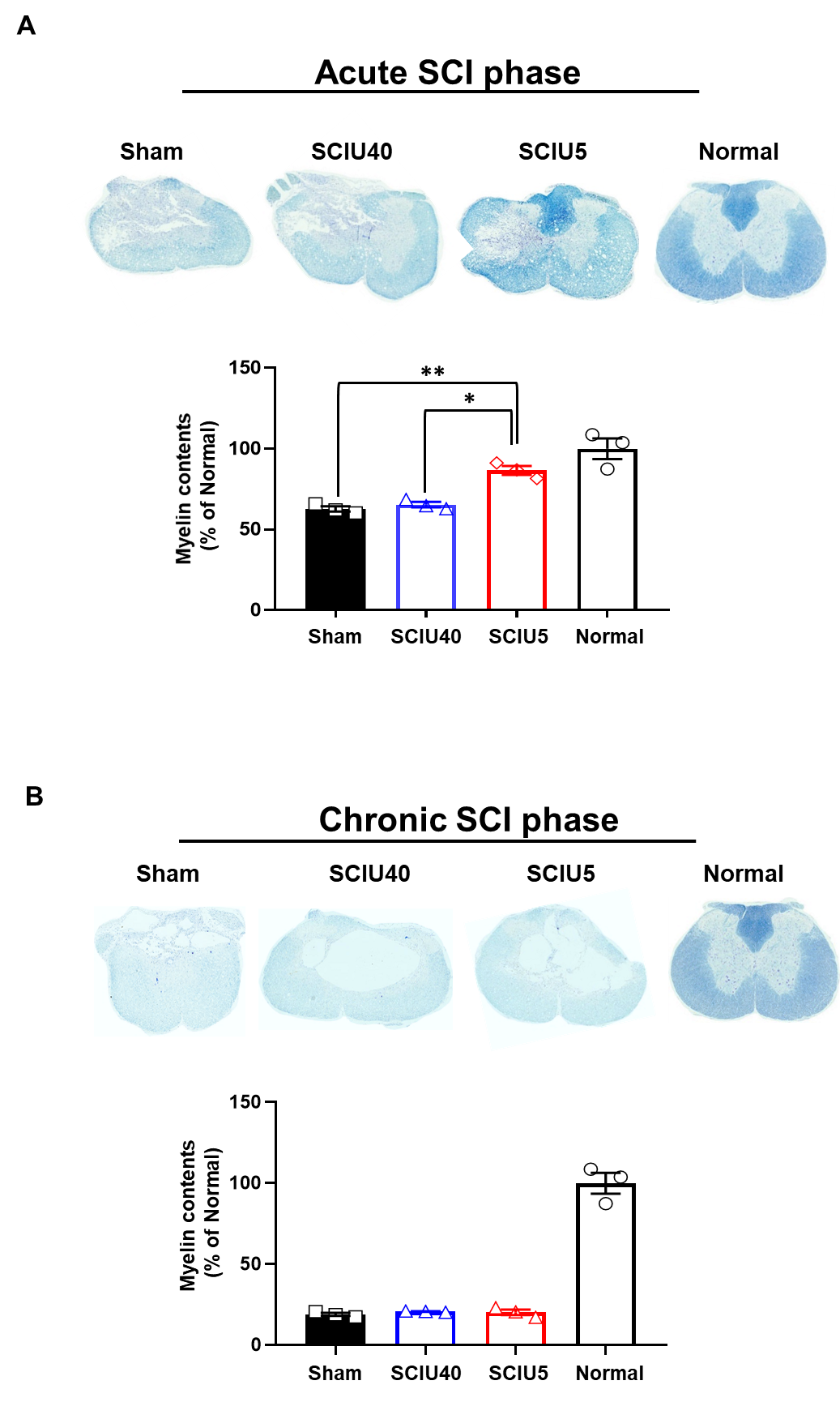


**Supplementary Figure 4. Axonal demyelination was protected by the suppression parameter ultrasound in acute phase injury-post 7 days, but not chronic phase injury-post 8 weeks.** (a) Representative LFB staining of the normal spinal cord tissue, SCI-sham, SCIU5, and SCIU40 at 7 days post-injury (upper panel) or 8 weeks post-injury (bottom panel). (b) Quantification data of the relative myelin density (% of the normal spinal cord) at 7 days post-injury (left bar graph) and 8 weeks post-injury (right bar graph). The quantitative values are shown as mean ± SD (n = 3). Statistical significance was analyzed by one-way ANOVA, followed by Tukey’s multiple comparisons. Scale bar: 500 μm.

***
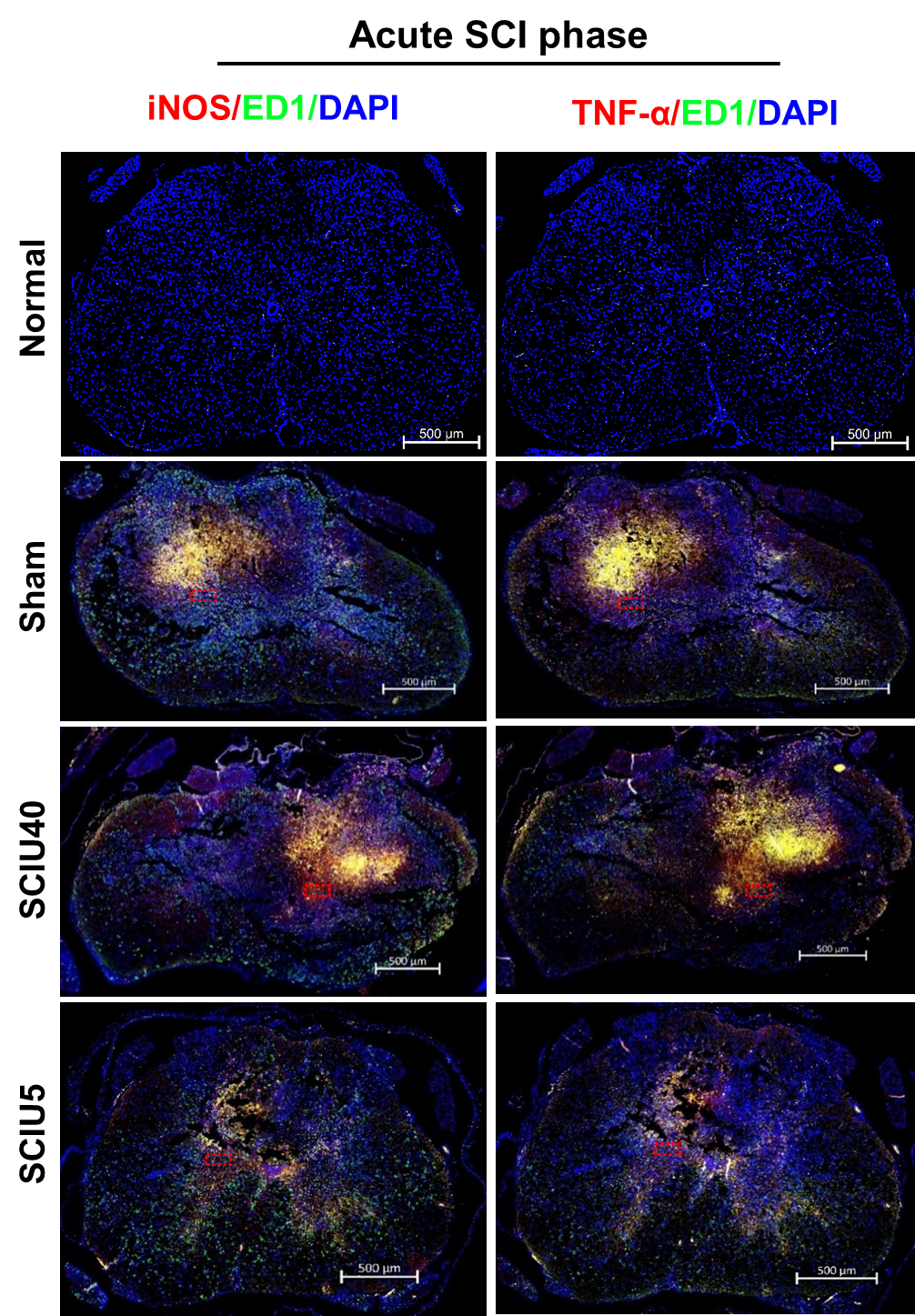
***

**Supplementary Figure 5. Representative whole tissue sections of SCI and normal control (uninjured tissue) stained for iNOS (red), ED-1 (green), and TNF-α (red).** The spinal cord sections were labeled with iNOS (red), ED-1 (green), and TNF-α (red) on day 7 after the treatment of rat models of SCI with US. Red dashed boxes indicate areas where immunofluorescent sections shown in Figure 2 were obtained. Scale bar represents 500 μm.


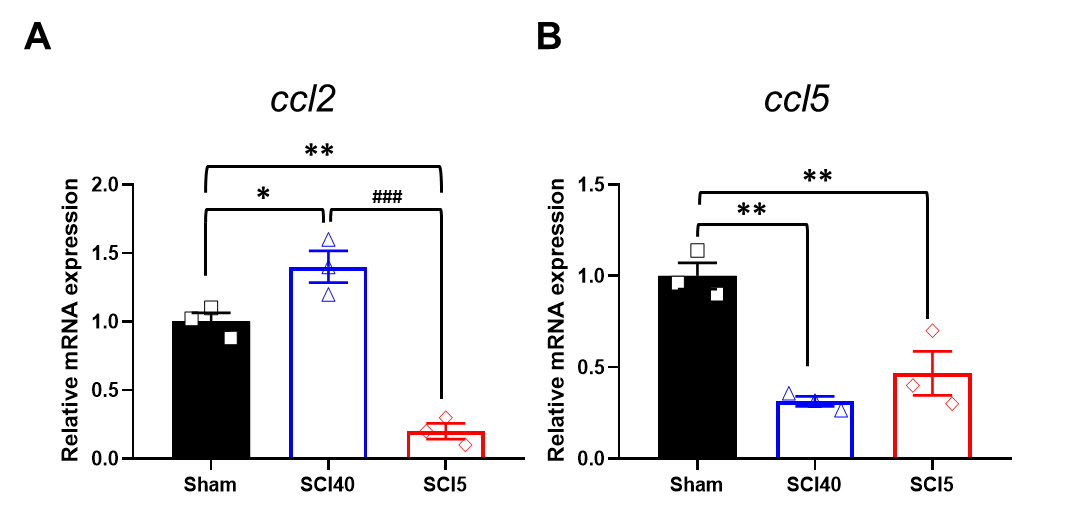


**Supplementary Figure 6. Real-time PCR analysis of gene expression of CCL2 and CCL5 at acute phase after injury**.

The injured spinal cord was isolated 7 days post-injury, and expression levels of CCL2 (a) and CCL5 (b) were assessed using quantitative real-time RT-PCR. The data are expressed as fold increase above Sham control normalized to gapdh. Data are shown as mean ± SD for n = 3 animals per group. Statistical significance was analyzed by one-way ANOVA, followed by Tukey’s multiple comparisons. Two-way ANOVA with Turkey's test for multiple comparisons was used for analyses. **P* < 0.05 and ***P* < 0.01 compared with the sham control, *^#^P* < 0.05 and *^##^P* < 0.01 compared with the SCIU5-treated group (n = 3/per group).


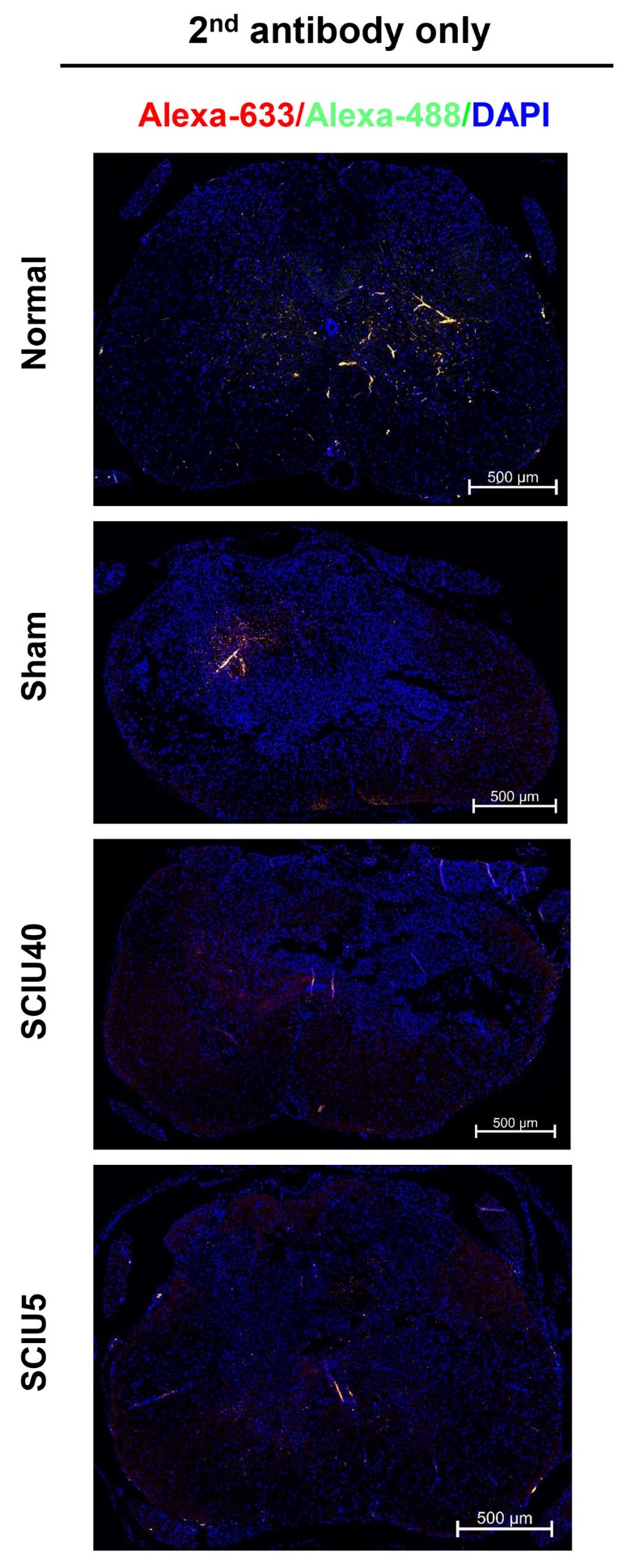


**Supplementary Figure 7. The fluorescence image of the SCI section and normal control stained with the secondary antibody.** The spinal cord sections were labeled with Alexa 488 (green) and Alexa 688 (red) on day 7 after treating the rat models of SCI with US. Scale bar represents 500 μm.


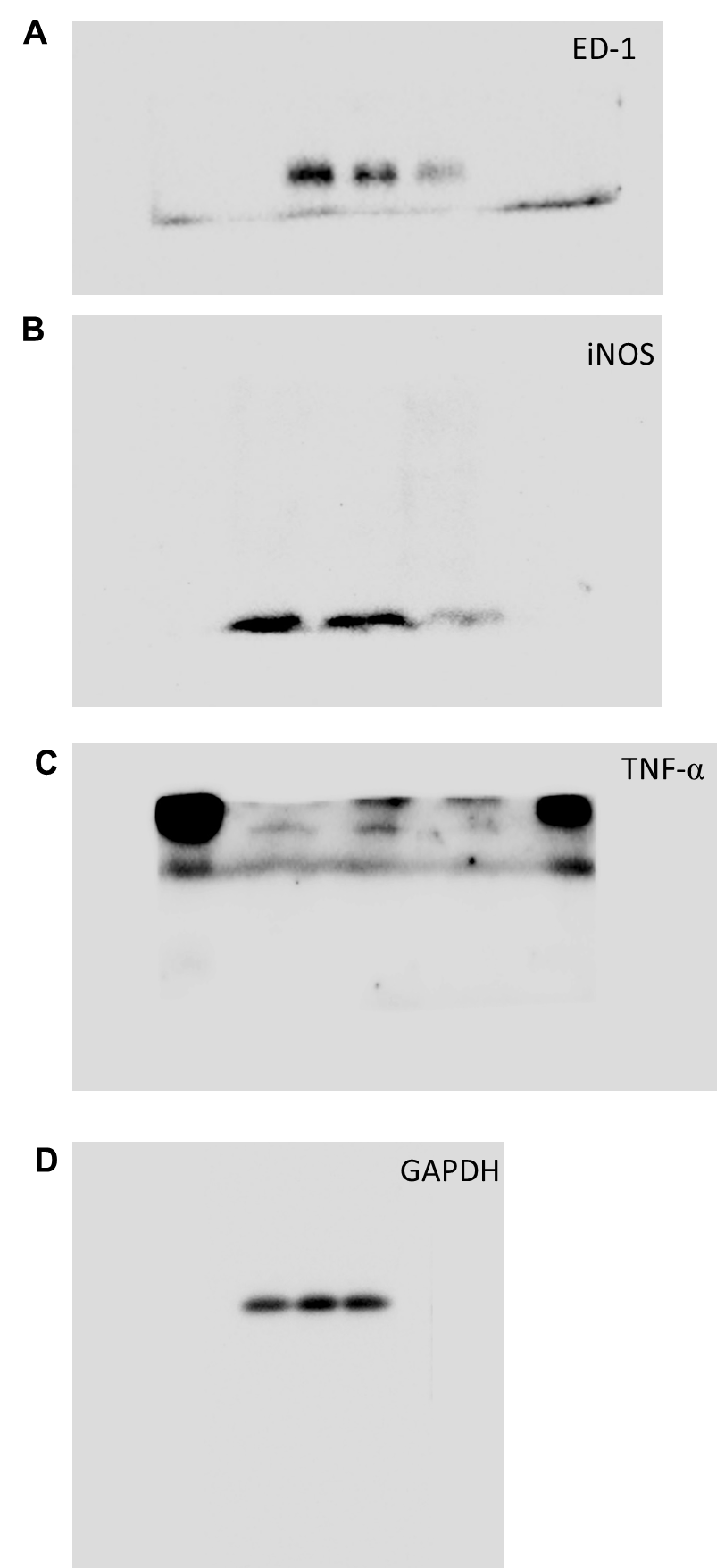


**Supplementary Figure 8. Full-length gel images of cropped gels.** Full-length gels for Figure 3**j**; (a) ED-1, (b) iNOS, (c) TNF-α and (d) GAPDH.
